# Rheological Behavior, Antimicrobial and Quorum Sensig Inhibition Study of an Argentinean Oregano Essential Oil Nanoemulsion

**DOI:** 10.3389/fnut.2020.569913

**Published:** 2020-10-09

**Authors:** Claudia Mariana Asensio, Patricia Raquel Quiroga, Ammar Al-Gburi, Quingron Huang, Nelson Rubén Grosso

**Affiliations:** ^1^Department of Food Science, School of Environmental and Biological Sciences, Rutgers, State University of New Jersey, New Brunswick, NJ, United States; ^2^Facultad de Ciencias Agropecuarias, Universidad Nacional de Córdoba (UNC), Instituto Multidisciplinario de Biología Vegetal (IMBIV), Consejo Nacional de Investigaciones Científicas y Técnicas (CONICET), Córdoba, Argentina

**Keywords:** oregano, nanoemulsion, quorum sensing, viscosity, viscoelasticity, oregano (*Origanum vulgare* L.)

## Abstract

In this study, Argentinean oregano essential oil (OEO) nanoemulsions (NEs) were developed. Four NEs were prepared: a control (CNE), EONE1 (10.6 mg EO/g NE), EONE2 (106 mg EO/ g NE), and EONE3 (160 mg EO/g NE) and tested for antimicrobial activity against *Staphylococcus aureus* ATCC 13565, *Listeria monocytogenes* Scott A, *Pseudomonas aeruginosa* ATCC 14213, and *Escherichia coli* O157:H7 using a broth microdilution assay and quorum sensing inhibition in a model using *Chromobacterium violaceum* ATCC 12472, where the production of violacein was quantified. The chemical composition of the EO was determined by gas chromatography–mass spectrometry. The average particle size (nm) and polydispersity index were monitored over 14 days at two different storage temperatures (4 and 23°C). A rheological behavior study was carried out using a dynamic shear rheometer, and flow curves, as well as viscoelastic properties, were determined. *E. coli* and *L. monocytogenes* were the most sensitive microorganisms to EONE (MIC of 2 and 5 mg/ml for EOEN3). Sub-MICs for NE were found at lower concentrations than those for pure EO. A significant reduction in violet pigment intensity and colorless coloration (*p* < 0.05) were observed at different NE concentrations concerning the control sample. The flow behavior index (*n*) decreased, and the consistency index (k) increased when the EO concentration was increased. CNE, EONE1, and EONE2 showed liquid-like behavior (G′ < G″) in the low-frequency region, whereas a solid-like behavior (G′ > G″) was observed in the high-frequency region, presenting a viscoelastic behavior, appearing as a wormlike micellar solution. For EONE3, a strong increase in both moduli was observed with increasing OEO concentration. The G′ was about one order of magnitude higher than the G″ over the whole frequency range, indicating the presence of a gel-like structure. The incorporation of EOs into an NE increased their stability, lowering the particle size, leading to a wormlike micelle with higher viscosity. Moreover, this NE had good antimicrobial activity and novel quorum-sensing inhibition activity. The results of this study indicated that Argentinean OEO NE could be used in a food system as a natural and stable antimicrobial agent.

## Introduction

Foodborne illnesses are a major concern for consumers, the food industry, and food safety authorities. In years past, an increase in the occurrence of disease outbreaks caused by pathogenic and spoilage microorganisms in foods has occurred (1). The misuse and mishandling of chemical antimicrobials have resulted not only in more tolerant and resistant viruses, bacteria, and parasites to chemical agents but also in hazards to human being's health, including respiratory allergies, and a rise in carcinogens and toxic substances. Moreover, consumers are concerned about the adverse effects of using synthetic antimicrobials and would prefer foods treated with safe and natural antibacterial agents (2).

Essential oils (EOs) are well-recognized as natural antimicrobial preservatives, are widely used as flavoring compounds, antimicrobial agents, and functional ingredients in food, and are classified by the US Food and Drug Administration as generally recognized as safe (3–5). Argentinian oregano essential oils (OEOs) were found to preserve the chemical, sensory, and microbiological qualities of ricotta cheese, cottage cheese, olive oil, roasted peanuts, fried peanuts, sunflower kernels, and hake burgers, among others (3, 6–10). However, their direct incorporation into foods is limited because of the hydrophobicity of these compounds and their difficulty interacting with microorganisms in aqueous media (11, 12). Moreover, due to their volatile nature, they can easily suffer degradation upon exposure to heat, pressure, light, and oxygen (13, 14).

In this context, nanoemulsions (NEs) are being used increasingly often to encapsulate, protect, and deliver lipophilic ingredients into liquid foods or minimally processed fruits and vegetables (12, 15). NE, owing to their subcellular size (20 and 200 nm), offers high thermodynamic stability, flocculation, and coalescence and increased distribution of the antimicrobial agent in food matrices, protecting it from deleterious interactions with food components and the environment (16). Furthermore, pH-, temperature-, and ionic-strength-sensitive compounds can be incorporated conveniently into food systems after nanoencapsulation (17).

The formation of NEs using medium-chain triacylglyceride (MCT) oils is often challenging due to their relatively low polarity, high interfacial tension, and high viscosity. It is difficult to prepare NEs from these oils using high-pressure homogenization methods because their high viscosity limits droplet disruption within the homogenizer. On the other hand, EOs have a relatively higher polarity, lower interfacial tension, and lower viscosity than MCT, which facilitates the formation of very small droplets by high-pressure homogenization (18, 19). This system is an effective method to achieve a constant release of bioactive compounds (10, 20) and allows to obtain NEs with smaller droplet sizes. Alexandre et al. (21) observed that increasing ginger EO concentration decreases the droplet size. The same behavior was found by Walker et al. (22) in NEs made with thyme EO, and these NEs were stable during storage. Preservation of food products with EO NE was reported. The addition of NEs with oregano EO protected hake (*Merluccius hubbsi*) burgers from deterioration, extending their shelf life (23). NE prepared with thyme EO and thymol effectively extended the shelf life of fresh pork (24). The antimicrobial activity of NEs based on different EOs was also investigated. Yazgan (25) demonstrated that sage EO and its nanoemulsified form could be used as a natural antimicrobial agent against food-related pathogens. NEs can be designed to form highly viscous or gel-like systems at much lower droplet concentrations than conventional emulsions.

Concentrated micellar solutions find applications in various industries (paint, chemical, pharmaceutical, drug delivery, and nanobiotechnology) and consumer products (home and personal care: detergents, hard surface cleaning, drain opening, perfumes, hair bleaching, skin cosmetics, shower gels, and sunscreens). The formation of entangled wormlike micelles (WLM) increases the viscosity of a solution and may even confer a certain viscoelastic property on it (26–29). The phenomenon of viscoelasticity can be induced by the addition of specific additives to some surfactants. Shear-thinning flow behavior often occurs at an increased surfactant concentration and in the presence of cosurfactants, additives, salts, or appropriate counterions (27, 28). Additives can compress the diffused electric double layer of the micellar interface, screen electrostatic repulsion between charged headgroups, and finally allow closer packing of the surfactant monomers into aggregates, resulting in the formation of a weak gel (27, 30).

Quorum sensing (QS) is a communication system that allows bacteria to monitor their population density through the production and sensing of small signaling molecules (31–33). *Chromobacterium violaceum* is a gram-negative bacterium that synthesizes a violet pigment (violacein) as a result of QS (34). Food deterioration, at least in part, is regulated by a mechanism of cell-to-cell communication (such as QS). Until now, only a few studies have developed food preservation techniques based on the anti-QS property of EOs (32). The search for an efficient QS inhibitor that can inhibit the spoilage of food products is a promising alternative (33). A stable NE with OEO that has antimicrobial and QS-inhibitory properties could be useful in food systems to avoid spoilage.

This study was performed with the following objectives: (i) to study the stability and to characterize the rheological properties of a stable NE with OEO; (ii) to determine the antimicrobial activity against food pathogens; and (iii) to evaluate the QS-inhibitory effects of OEO NE.

## Materials and Methods

### Materials

Leaves and flowers of *Origanum vulgare* ssp. *hirtum* (clone Criollo) were purchased (crop 2015) on a farm located in Villa Dolores (Córdoba, Argentina).

Neobee 1053 MCT was a gift from Stepan Co. (Northfield, USA). Alcolec PC75 (phosphatidylcholine enriched) soy lecithin was obtained from American Lecithin Co. (Oxford, USA). All other chemicals were purchased from Sigma-Aldrich (St. Louis, USA). Deionized water was obtained from a Milli-Q water purification system (Millipore Co., Bedford, USA) and used in all experiments. Culture media were purchased from BD Bacto™ (New Yersey, USA).

### Essential Oil Extraction and Analysis

Dried leaves and flowers were hydro-distilled for 2 h in a Clevenger-type apparatus with a separated extraction chamber. The EOs were kept in dark flasks at −18°C in a freezer. The EO was analyzed with a Perkin Elmer Clarus 600 gas chromatography–mass spectrometry (Shelton, Connecticut, USA) coupled with an ion trap mass detector (MS) and non-polar capillary column Elite-5 MS (methylpolysiloxane, 5% phenyl, 30 m, 0.25 mm id, and 0.25 mm coating thickness). The compounds were identified by comparing their mass spectra with those from the literature (35) and the National Institute of Standards and Technology (2.0) library (NIST 2.0). The main components were further identified by the co-injection of authentic standards (Sigma® St. Louis, MO, USA). The quantitative composition was obtained by peak area normalization, and the response factor for each component was considered equal (5).

### Nanoemulsion Preparation and Physical Stability Characterization

An aqueous solution of soy lecithin was prepared to disperse the dried lecithin powder in deionized water at room temperature and stirring for 30 min. The oil phase consisted of MCT and *Origanum* EO mixed at different ratios (15:1, 1.5:1, and 1:1 w/w). The final NE composition was 81% water, 3% soy lecithin, and 16% oil phase. Both phases were premixed with a high-speed homogenizer (Ultra-Turrax T25 IKA Works Inc., Wilmington, NC, USA), equipped with an S25 N18 G rotor operated at 12,000 rpm per 3 min at room temperature. These coarse emulsions were finely homogenized with a high-pressure homogenizer (EmulsiFlex-C3, Avestin Inc., Ottawa, Canada) for six cycles at a pressure of 150 MPa. Four NEs were prepared: CNE, EONE1, EONE2, and EONE3. After homogenization, samples were stored in 20-ml glass vials (Supelco Analytical) with a screw cap (PTFE/silicone septum, Supelco Analytical) covered with aluminum foil in two different storage conditions: room temperature (RT) (23°C) and fridge (F) (4°C), and stored for 14 days.

#### Particle Size and Polydispersity Index

The average particle size (mean diameters, nm) and polydispersity index (PDI) of NEs were monitored over 14 days using a dynamic light scattering instrument (Brookhaven BIC 90 plus) equipped with a Brookhaven BI-9000AT digital correlator (Brookhaven Instrument Corp, New York, NY) to evaluate the stability. Samples were diluted 1:100 using deionized water to prevent multiple scattering effects. All measurements were performed in triplicate at a fixed scattering angle at 25°C. The light source of the particle size analyzer was a solid-state laser operating at 658 nm with 30 mW power. The mean diameters of emulsions were determined by cumulant analysis of the intensity–intensity autocorrelation function, G (q, t).

### Rheological Behavior Study

The rheological characteristic study provides information about the behavior of the fluid, shear-thinning or shear-thickening nature. Viscosities of formulated NEs from MCT surfactants were measured with a dynamic shear rheometer (Discovery Hybrid Rheometer, TA Instruments, DE, USA) equipped with a cone-plate geometry with a cone diameter of 60 mm. Flow curves were determined at 25°C with two consecutive continuous shear rate ramps from 0.50 to 150 s^−1^. The apparent shear viscosity at a fixed shear rate (100 s^−1^) was reported. The viscosity curves were analyzed using the power-law mathematical model. The value of *n* defines the nature of the fluids. If *n* < 1 → shear-thinning nature, *n* = 1 → Newtonian fluid nature, and *n* > 1 → shear-thickening nature (36).

#### Pseudo Plastic Nature

The following equation describes the pseudoplastic behavior:

(1) $$\begin{array}{l} \tau =k\times {\overset{∙}{\gamma }}^{n} \end{array}$$

where τ is the shear stress, k is the consistency index, *n* is the flow behavior index, and $\overset{∙}{\gamma }$ is the shear rate. Values of *n* and k were calculated, taking the log on both sides of Equation (1) and plotting log τ vs. log $\overset{∙}{\gamma }$. The apparent or effective viscosity (μ) along with *n* and k values was determined by fitting the experimental data to the following equation:

(2) $$\begin{array}{l} \mu =k{\overset{∙}{\gamma }}^{n-1} \end{array}$$

although the power-law model is a good descriptor of fluid behavior across a shear rate range up to which coefficients are fitted, due to its simplicity with more versatility than the Bingham plastic model, it is widely practiced (37).

#### Viscoelastic Properties Analysis

Viscoelastic property study provides information about the elastic and viscous nature of fluids and involves the determination of real, storage, or elastic modulus (G′), and imaginary, loss or viscous modulus (G″) as a function of angular frequency (ω) (37). Strain sweep experiments (data not shown) were performed to determine the linear viscoelastic regions of the samples at 25°C and a constant frequency of 1 Hz, with a strain % in the range of 0.1–100%. Frequency sweep tests were performed using a strain amplitude of 0.4 (within the linear viscoelastic regions) over an angular frequency range of 0.1–100 rad s^−1^. Finally, G′ and G″ moduli curves were plotted against angular frequency. If G′ > G″, fluid behaves as solid or gel, and for G′ < G,” fluid acts as liquid or viscous (36, 37).

### Antimicrobial Activity

#### Microbial Strains

The antimicrobial activity of the EO and its NEs was tested against: *S. ureus* ATCC 13565, *Listeria monocytogenes* Scott A (*L. monocytogenes*), *Pseudomonas aeruginosa* ATCC 14213, and *Escherichia coli* O157:H7. The cultures were obtained from the Department of Food Science, Rutgers University, culture collection (New Brunswick, NJ, USA). From a frozen stock (−80°C), bacteria were inoculated into trypticase soy agar plates (TSA, Becton Dickson and Co., Cockeysville, MD, USA) and propagated under aerobic conditions at 37°C for 24 h. After the incubation, one colony of each bacterial strain was transferred separately to test tubes with trypticase soy broth (TSB) and incubated at 37°C with agitation for 18–24 h. For broth microdilution assay, the bacterial growth suspensions were further diluted in fresh TSB medium to achieve 10^6^ colony forming units (CFU)/ml (38).

*C. violaceum* ATCC 12472 was grown in Luria–Bertani (LB) broth (ACROS, Miller, NJ) at 26°C for 48 h aerobically. *P. aeruginosa* ATCC 14213 was aerobically grown in LB broth at 37°C for 24 h and used as a positive control for QS inhibition in gram-negative bacteria (39).

#### Determination of Antimicrobial Activity

Antimicrobial activity was performed using a broth microdilution assay (40). Briefly, EO (first diluted in dimethyl sulfoxide) and NEs were 2-fold diluted with fresh TSB in a 96-well tissue culture plate (Falcon, Corning Incorporated, Corning, NY, USA). The final volume of the antimicrobial diluted into the broth was 100 μl in each well. The overnight cell culture was diluted in TSB to the final 5 × 10^6^ CFU ml^−1^ (the number of bacterial cells was confirmed by the spot-plate method). From the diluted bacterial cells, 100 μl was transferred in the wells containing predetermined concentrations of antimicrobials. Plates were incubated under aerobic conditions at 37°C for 24 h. Mineral oil (Sigma-Aldrich chemical, St. Louis, MO, USA) was added (75 μl) to each well to avoid evaporation. The optical density readings of the microorganism at 595 nm were tracked using a microplate reader (Model 550, Bio-Rad Laboratories, Hercules, CA). The minimum inhibitory concentration (MIC) was defined as the lowest concentration of antimicrobial agent that produced no visible growth after overnight incubation (38, 39). Each experiment was performed three times in duplicates.

### Quorum Sensing Inhibition Assay

This assay was performed according to Algburi et al. (39). Briefly, the overnight-grown cells of *C. violaceum* were diluted in fresh LB broth to achieve 10^6^ CFU/ml. NE-containing EOs were serially 2-fold diluted with LB into a 48-well microplate (BD, Franklin Lakes, NJ). A bacterial suspension (500 μl) (10^6^ CFU/ml) was mixed with 500 μl of LB broth and 500 μl of the NE dilution. Once the samples were prepared, the plate was aerobically incubated at 26°C without shaking for 48 h. The cell-free supernatant (CFS) of *P. aeruginosa* grown in LB was used as a control, preventing violacein production by *C. violaceum*.

#### Quantification of Violacein Production

After incubation, 750 μl from each well (test and control wells) was transferred to a 1-ml centrifuge tube and centrifuged at 8,000 g for 5 to collect violacein and the producer cells. The supernatants were discarded, and the pellets were vigorously vortexed with 750 μl of 100% dimethyl sulfoxide to dissolve the insoluble violacein. The samples were centrifuged again at 8,000 g for 5 min to precipitate *C. violaceum* cells. To evaluate violacein production, 200 μl of violacein-containing supernatant was added into a 96-well microplate (Falcon, Corning Inc., Corning, NY) in triplicate, and the optical density at 585 nm (OD585) was measured using a plate reader (Model 550, Bio-Rad Laboratories, Hercules, CA). To ensure that the QS inhibition occurred without NE killing the targeted microorganisms by sub-NE MICs, the precipitated bacterial cells were resuspended in 750 μl of DW (pH 7.0), and the absorbance was measured at OD600. The ODs of cells treated with sub-MICs of NE were compared against the non-treated cells (positive control). The control value was set to 100% violacein production (39).

Violacein inhibition (%) = 100-((OD_585_ Sample - OD_585_ PFSC Control) ^*^ 100/OD_585_ Control).

The 95 (%) violacein inhibition concentration was calculated considering the regression equations (*R*^2^ ≥ 0.8) of each treatment obtained by plotting violacein inhibition (%) against samples' concentrations.

### Statistical Analysis

The experiments were carried out three times, and results were expressed as mean ± standard deviations. Normal distribution was tested with a Shapiro–Wilk test. Analysis of variance (ANOVA, α = 0.05) and Fisher's least significant difference multiple range test were performed to determine significant differences between means. Data were analyzed using the InfoStat software, version 2019 (41).

## Results and Discussion

### Essential Oil Composition

The chemical composition of EO from *O. vulgare* ssp. *hirtum* clone Criollo is shown in Table 1. The major compounds were terpinolene (18.62 g/100 g), thymol (16.87 g/100 g), γ-terpinene (15.08 g/100 g), and ortho-cymene (11.08 g/100 g). A similar composition was reported previously (5, 23, 42). This clone was shown to have higher levels of the bioactive phenol thymol than any other OEO from Argentina (5, 43). Moreover, previous studies reported that EOs of both *O. vulgare* ssp. *hirtum* clones (Criollo and Cordobes) were more active in *in vitro* antimicrobial tests than other tested OEOs (3, 5).

**Table 1:** Oregano clone Criollo essential oil chemical composition analyzed by gas chromatography–mass spectrometry.

	**RT**	**Compound**	**EO**^**‡**^	
		**(g/100 g)**	**$\bar{X}$**	**SD**
1	8.41	α-Phellandrene	1.02	0.04
2	8.53	α-Pinene	0.76	0.01
3	8.72	Camphene	0.34	0.01
4	8.90	β-Pinene	3.8	0.02
5	8.97	3-Octanone	1.65	0.02
6	9.06	β-Myrcene	0.28	0.01
7	9.34	α-Terpinene	4.00	0.05
**8**	**9.41**	**0rto-cymene**	**11.08**	**0.16**
9	9.51	β-trans-Ocimene	1.02	0.04
**10**	**9.74**	**γ-Terpinen**	**15.08**	**0.03**
11	9.88	cis Sabinene hidrate	2.52	0.16
12	10.02	β-cis-Ocimene	0.88	0.03
**13**	**10.20**	**Terpinolene**	**18.62**	**0.53**
14	11.00	Borneol	0.51	0.4
15	**11.12**	**4-Terpineol**	**9.28**	**0.22**
16	11.47	α-Terpineol	2.28	0.1
17	11.51	Thymol methyl ether	1.02	0.03
18	11.98	Carvacrol methyl ether	0.31	0.05
**19**	**12.08**	**Thymol**	**16.87**	**0.78**
20	13.06	Carvacrol	0.83	0.09
21	14.03	Caryophyllene	1.77	0.03
22	14.33	Germacrene D	0.85	0.01
23	15.01	γ-Gurjunene	1.14	0.03
24	15.09	δ-Cadinene	1.01	0.01
25	15.12	Aromadendrene, dehydro-	2.29	0.02
26	15.19	Lanceol, cis	1.14	0.02

‡ *Only those compounds with amount higher than 0.3 g/100 g are presented. Letters and numbers in bold indicate main compounds*.

### Nanoemulsion Characterization

#### Physical Stability of Nanoemulsions

The mean particle sizes after homogenization were 150.77, 131.7, 89.17, and 74.7 for CNE, EONE1 (15:1 10.6 mg EO/g NE), EONE2 (1.5:1 106 mg EO/g NE), and EONE3 (1:1 160 mg EO/g NE), respectively. From these data, it was concluded that EONE3 had the smallest droplets and the lowest PDI values. Therefore, EONE3 was the most stable. These results were confirmed after 14 days in storage; droplet sizes and PDI values remained the lowest for EONE3 (Table 2).

**Table 2:** Means and standard deviations (*n* = 3) of effective diameter (ED) and polydispersity index (PDI) of control (CNE) and essential oil-containing nanoemulsions (EONE1, EONE2, and EONE3).

		**Day 0**					**Day 14**				
**Sample**	**Temp**				^**†**^	^**‡**^				^**†**^	^**†**^
ED											
CNE	F	150.77	±	0.06	D	1	148.00	±	2.90	F	1
	RT	150.77	±	0.06	D	2	111.75	±	2.65	D	1
EONE1	F	131.70	±	0.20	C	1	127.57	±	3.02	E	1
	RT	131.70	±	0.20	C	2	98.10	±	5.87	C	1
EONE2	F	89.17	±	5.71	B	2	73.83	±	5.84	B	1
	RT	89.17	±	5.71	B	2	64.17	±	2.25	A	1
EONE3	F	42.80	±	3.00	A	1	62.60	±	0.20	A	2
	RT	42.80	±	3.00	A	1	62.90	±	0.20	A	2
PDI											
CNE	F	0.307	±	0.002	A	1	0.314	±	0.01	A	1
	RT	0.307	±	0.002	A	1	0.356	±	0.01	B	2
EONE1	F	0.292	±	0.003	A	1	0.332	±	0.02	A	2
	RT	0.292	±	0.003	A	1	0.334	±	0.01	A	2
EONE2	F	0.288	±	0.049	A	1	0.322	±	0.03	A	1
	RT	0.288	±	0.049	A	1	0.320	±	0.03	A	1
EONE3	F	0.278	±	0.007	A	1	0.316	±	0.01	A	2
	RT	0.278	±	0.007	A	1	0.310	±	0.02	A	1

† Different letters in each column indicate significant differences between samples (ANOVA, DCG test, alpha 0.05).

‡ *Different numbers in each row indicate significant differences between storage temperatures for each sample (ANOVA, DCG test, a = 0.05)*.

The droplet size decreased, as the proportion of OEO in the NE increased. Similar behavior was found for NEs prepared with ginger EO (21) and lemon EO (22). The size of the droplets produced during high-pressure homogenization typically decreases, as the oil phase viscosity and interfacial tension decrease; this facilitates droplet disruption (44).

It is difficult to prepare NEs from MCT oils (18, 19). With the addition of EOs as cosurfactants, it is expected the formation of very small droplets during homogenization when the EO concentration increases, as in this case. The droplet size of the EONE stored at 4 and 23°C tended to decrease during the storage, except for EONE3. Similar behavior was found by Pongsumpun et al. (45). They observed that the droplet size of the cinnamon EO NE stored at 4 and 30°C decreased during the first 60 and 30 days of storage, respectively. Afterward, the size of the droplets tended to increase, probably due to the coalescence of the emulsion droplets. It could be possible that droplet sizes of EONEs would increase if storage time increases.

The EONEs had no visible creaming or phase separation (data not shown) during the storage at both tested temperatures. Therefore, the NEs had good stability in terms of droplet size under the tested temperatures and storage time.

### Rheological Behavior Study

#### Viscosity

Table 3 presents the values of flow behavior (*n*) and consistency (k) indexes for the prepared NE samples. The power-law model describes with high accuracy the flow curves of prepared NEs. As seen from Table 3, the values of *n* for samples EONE2 (0.8375) and EONE3 (0.7542) were <1; these NEs showed non-Newtonian or shear thinning behavior. EONE1 (1.0545) showed Newtonian behavior, and CNE (1.1553) demonstrated shear thickening behavior. The values of *n* decreased, and those of *k* increased, as the EO concentration increased.

**Table 3:** Consistency coefficient (k) and flow behavior index (*n*) of tested nanoemulsions.

	**CNE^**†**^**	**EONE1^**†**^**	**EONE2^**†**^**	**EONE3^**†**^**
*k* (Pa. s)	0.0013	0.0037	0.0186	0.0537
*N*	1.1553	1.0545	0.8375	0.7542
R^2^	0.9992	0.9998	0.9998	0.9994

† *Treatments: control (CNE) and essential oil-containing nanoemulsions at different concentrations (EONE1, EONE2, and EONE3)*.

The viscosity of a micellar solution with a hydrophilic surfactant increases gradually when a lipophilic surfactant is added and increases steeply above a certain level due to the formation of WLM (28). Mitrinova et al. (26) found that some terpenes increased the solution viscosity and led to shear thinning behavior. Such non-Newtonian solution behavior was associated with the formation of entangled WLM in the solutions (26). Polar terpenes could solubilize in the palisade layer, changing the curvature of the micellar surface and thus increasing the solution viscosity (26, 28, 46). Terpenes from the oregano clone Criollo EO could act as cosurfactants, solubilizing in the palisade layer. Depending on their molecular structure and polarity, they could increase NE viscosity, leading to shear thinning behavior and probably forming WLM.

#### Viscoelastic Property Analysis

**Figure 1:**
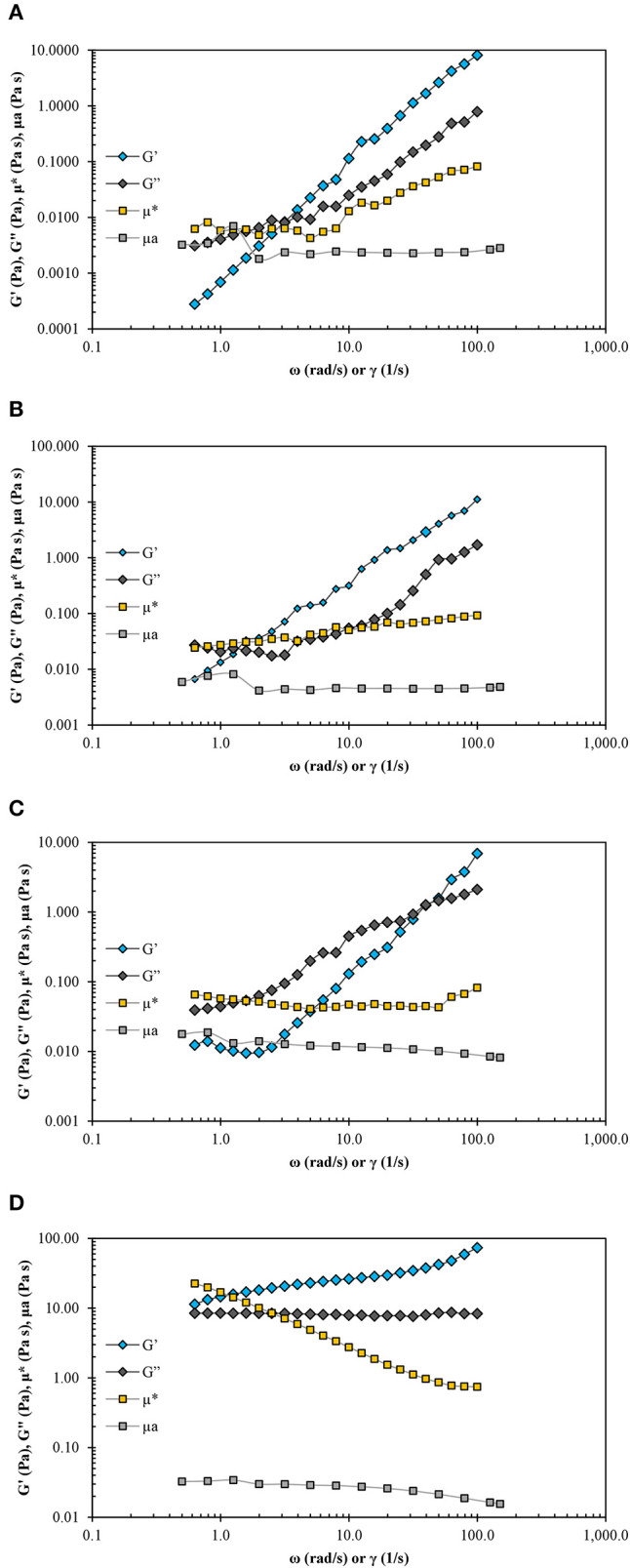
Storage (G′) and loss (G″) modulus, complex viscosity (μ*), and apparent viscosity (μ_a_) of nanoemulsion: **(A)** CNE, **(B)** EONE1, **(C)** EONE2, and **(D)** EONE3 as a function of the angular frequency (ω) or shear rate $\left(\overset{∙}{\gamma }\right)$.

The variation in dynamic moduli (G′ and G″) and complex viscosity (μ^*^) as a function of the angular frequency (ω) and the variation in apparent viscosity (μ_a_) as a function of the shear rate $\left(\overset{∙}{\gamma }\right)$ for NE samples are shown in Figure 1.

From dynamic rheological tests in the linear viscoelastic range, the storage modulus G′ and the loss modulus G″ were obtained. G′ value is a measure of the deformation energy stored in the sample during the shear process, representing the elastic behavior of a sample. In contrast, the G″ value is a measure of the deformation energy used up in the sample during the shear and lost to the sample afterward, representing the viscous behavior of a sample (36). CNE, EONE1, and EONE2 showed liquid-like behavior (G′ < G″) (Figures 1A–C) in low-frequency regions, whereas solid-like behavior (G′ > G″) was observed in high-frequency regions. This is a typical viscoelastic behavior shown by WLM solutions (28).

In EONE3, the storage modulus (G′) was above the loss modulus (G′′) over the whole frequency range analyzed (Figure 1D). When the G′ and G″ curves do not cross over the whole frequency range, this indicates that a gel-like structure is present (36, 47), as in this case. The decrease in μ^*^ (complex viscosity) in EONE3 (Figure 1D) confirmed the shear-thinning nature of this NE. Moreover, values of μ^*^ were greater than μ_a_ for all magnitudes of shear rates and oscillatory frequencies; this NE sample did not behave as a true gel. In Figure 1D, complex viscosity as a function of angular frequency lay above the curve of apparent viscosity as a function of the shear rate. This behavior is typical for a weak gel. Such systems do not obey the Cox–Merz rule, which states that the frequency dependence of complex viscosity (μ^*^) and the shear rate dependence of apparent viscosity (μ_a_) are similar at the same corresponding values of frequency (measured in radians s^−1^) and shear rate (s^−1^) (30).

**Figure 2:**
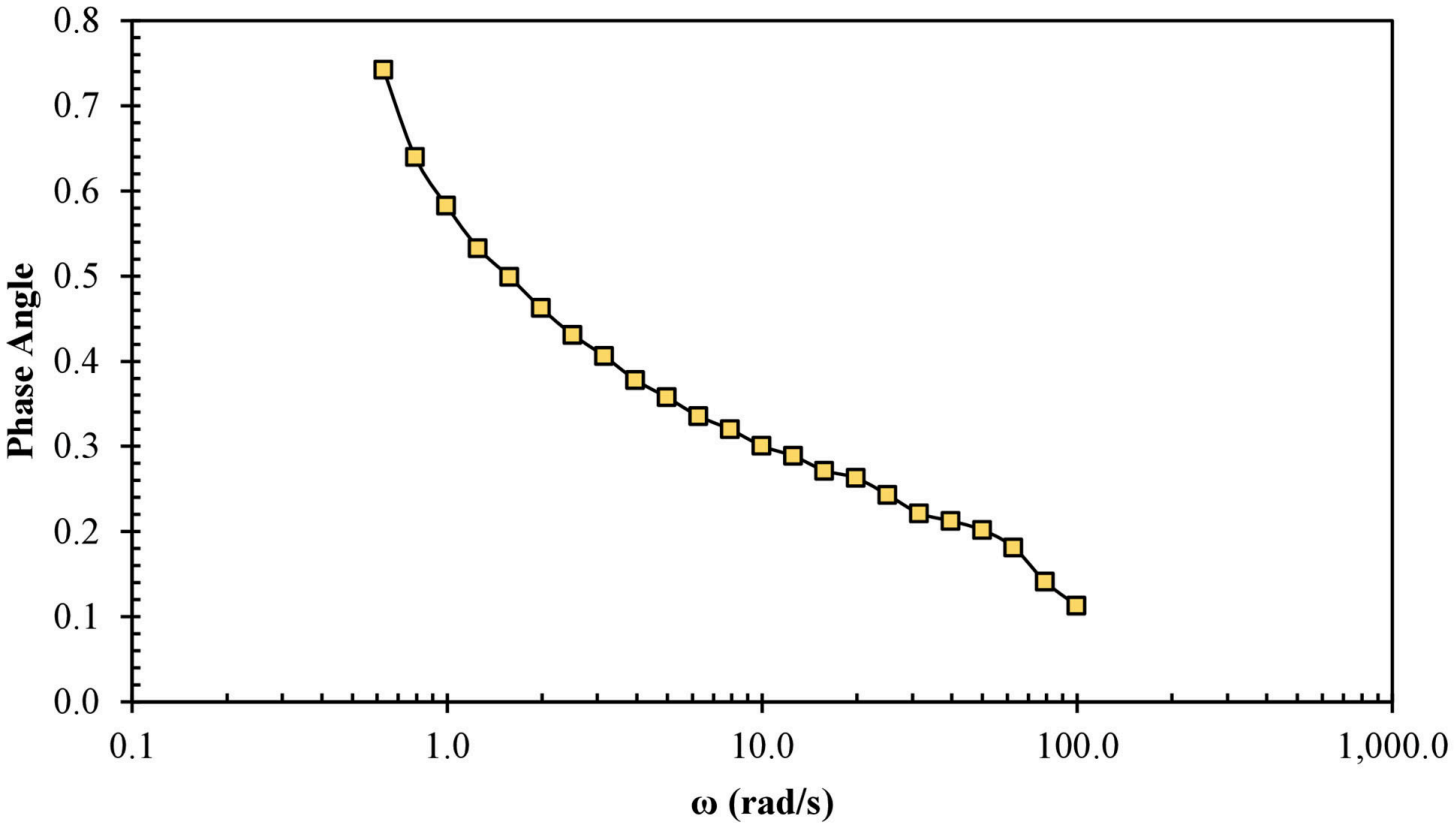
Gel network behavior of EONE3: changes of phase angle as a function of the angular frequency.

In the plot of frequency dependence as a function of the phase angle of EONE3 (Figure 2), it was observed that the phase angle decreased, as the frequency increased over the whole range of frequencies, which indicates that in EONE3, there was no damage to the gel network. Moreover, this suggested that EONE3 did not behave as a true gel, as described by Mezger (36). Terpenes, when solubilized in the palisade layer, could decrease the repulsive forces on the micellar surface and finally allow closer packing of the surfactant monomers in the aggregates, resulting in the formation of a weak gel, as in this NE (26, 28, 36).

### Antimicrobial Activity

**Figure 3:**
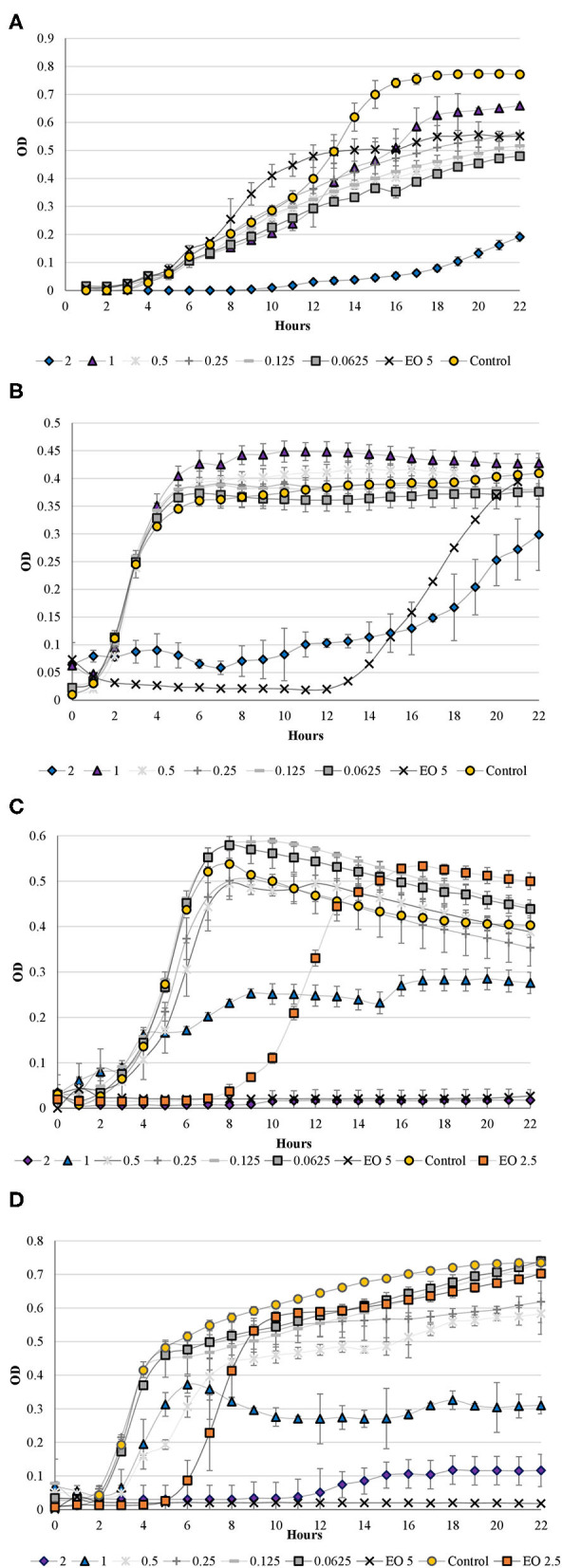
Antimicrobial activity of oregano essential oil (EO) (5 and 2.5 mg/ml) and oregano nanoemulsion (concentrations: 0.0625 to 2 mg/ml) against: **(A)** *P. aeruginosa*, **(B)** *S. aureus*, **(C)** *E. coli*, and **(D)** *L. monocytogenes*.

The broth microdilution method was used to determine the MICs and sub-MICs of OEO and its NE against *P. aeruginosa, S. aureus, L. monocytogenes*, and *E. coli* (Figures 3A–D). The sub-MICs of OEO NEs were identified for use in the quorum-sensing inhibition assay. EO concentrations as high as 5 mg/ml could not inhibit the growth of *P. aeruginosa* and *S. aureus* (Figures 3A,B). However, EONE3 had a greater inhibitory effect against these microorganisms than the EO, and sub-MICs were also identified.

The antimicrobial or other biological activities of OEOs has been deeply studied; there is a direct correlation between chemical composition and biological properties (3, 5, 23). The antimicrobial effects are related to phenolic compounds, monoterpenes hydrocarbon, total monoterpenes, and sesquiterpenes. Argentinean OEOs are rich in acyclic compounds and sesquiterpenoids. In previous studies, terpenes as p-cymene and thymol were mainly responsible for the antimicrobial activity. Those results revealed that not only gram-positive bacteria but also gram-negative bacteria showed sensitivity to these oils (3, 6). Thymol acts as a transmembrane carrier of monovalent cations by exchanging their hydroxyl proton for another ion. Cyemene does not have this property but acts synergistically, expanding the membrane. Terpineol has a hydroxyl group, but it does not possess high antimicrobial activity, probably because of the absence of delocalized electron system of double bonds (48, 49). Moreover, the synergistic action taking place among the components of an EOs has greater antimicrobial activity than the major components alone (5, 49, 50).

NEs interact with the lipids of microorganisms to cause cell death (51). The electrostatic attraction can improve their chances of combining with charges on the pathogen surface. When NEs combine with the microorganism, they discharge some portion of their interior contents resulting in cell lysis (52). Emulsification enhanced the dispersibility of EO in aqueous solution, and its physicochemical stability, therefore, increased its antimicrobial activity (53).

The obtained results show that *E. coli* (MIC of 5 and 2 mg/ml EO and EONE3) and *L. monocytogenes* (MIC 5 mg EO/ml) (Figures 3C,D) were the most sensitive to both OEO and NE. The concentration of EO in the NE was 160 mg/G, 6.25 times lower than that of pure EO. Moreover, sub-MICs of the NE were found for both microorganisms at lower concentrations than for pure EO. It was observed for *E.coli* that 5 mg/ml of OEO and 2 mg/ml EONE3 inhibited the growth; in the same way, 2.5-mg/ml EO inhibited the growth of bacteria for 8 h, and 1 mg/ml of EONE3 allowed the growth of bacteria but at one half the concentration than the control sample. Regarding the gram-negative *P. aeruginosa*, no MIC was found. However, 2-mg/ml EONE3 inhibited the growth of cells for 9 h, and an OD much lower than the OD registered for the control sample was observed at the end of the study. *P. aeruginosa* has various virulence mechanisms and a diverse metabolic capacity, which makes it resistant to antibiotics because of its impermeable outer membrane, efflux capabilities, tendency to colonize surfaces in a biofilm form, and ability to acquire and maintain antibiotic plasmids (54). Gram-negative bacteria have a complex structure of membrane phospholipids, proteins, and lipid-based peptidoglycan. The presence of an outer hydrophilic membrane embedded with lipopolysaccharide molecules on gram-negative bacteria serves as an effective protective barrier toward macromolecules and hydrophobic compounds (55, 56). However, it was demonstrated that highly lipophilic compounds penetrate easily through the outer membrane of several bacteria (49). The peptidoglycan layer of the gram-positive *L. monocytogenes* is not as effective as a barrier against antimicrobial agents. In this research, the antimicrobial influence of pure EO (MIC 5 mg/ml) was observed. However, a complete inhibitory effect was also observed for EONE3 (2 mg/ml) for 12 h of incubation, and substantially lower growth of cells was observed in the treatment with 1-mg/ml EONE3. In a different study, the MIC and MBC of an OEO for *L. monocytogenes* and *E. coli* were 50 μl/ml, whereas those of the OEO NE were 10 and 15 μl/ml for *L. monocytogenes* and *E. coli*, respectively. EO NEs were observed to be more effective against *E. coli* and *L. monocytogenes* than the non-encapsulated EOs applied directly (57), as it was found in this study. Nanoencapsulation was observed to reduce the MIC and MBC of oregano, rosemary, and cinnamon EOs by an average of 50% (57). In a different study, a blended clove/cinnamon EO NE showed higher antimicrobial activity against *E. coli, Bacillus subtilis, S. typhimurium*, and *S. aureus* than their individual non-NE counterparts, even at far lower concentrations (58). The antimicrobial effects of thyme NEs against foodborne pathogens were significantly higher (*P* < 0.05) than the pure EO (59). On the contrary, in a different study where sage EO and its NEs were tested as antimicrobials against fish spoilage bacteria, it was found that pure sage EO had more effectiveness than the NE form (25). This confirms that the bioactivity of NEs based on EO varies with droplet size, emulsion formulation, EO chemical composition, viscosity, and microbial strain (60).

### Quorum-Sensing Inhibitory Effects of Essential Oil Nanoemulsions

Inhibition of violacein production of *C. violaceum* is commonly used as an indicator of QS inhibition in gram-negative bacteria (31, 32). The QS mechanism in *C. violaceum* ATCC 12472 is controlled by the CviI/CviR system (Luxl/LuxR homologs), correlated with the production of the purple pigment violacein in response to threshold concentrations of the autoinducer N-hexanoyl homoserine lactone (61). EONEs at sub-MICs were tested. A significant reduction in the violet pigment and colorless colorations (*p* < 0.05) were observed at different NE concentrations. The concentrations of EO in the NE that inhibited 97.4–88.9% of violacein production ranged from 0.125 to 0.039 mg EO/ml. Similar concentrations of pure EO (0.078 mg/ml and 0.039 mg EO/ml) inhibited 94.3 and 66.67% violacein coloration. These results demonstrated that when the concentration of EO decreases, violacein inhibition also decreases but differently depending on if the EO is pure or in the NE. No significant differences in violacein inhibition were registered between the tested NE concentrations (*p* > 0.05); all the tested concentrations produced a similar effect on violacein coloration. Similar results were observed when thyme EO, carvacrol, and thymol caused 90, 80, and 78% inhibition of violacein synthesis, respectively, after 72 h of culturing (62). The inhibition of violacein production by *C. violaceum* ATCC 12472 by carvacrol was 40% at 0.7 mM (equal to 0.105 mg/ml) (63). In a different study, cumin oil NE exhibited 42.2% inhibition of violacein production at 40 μl/ml, whereas pepper oil showed 15.8% inhibition at 50 μl/ml. In that case, cumin EO NE showed higher bioactivity than pepper EO NE (64).

**Figure 4:**
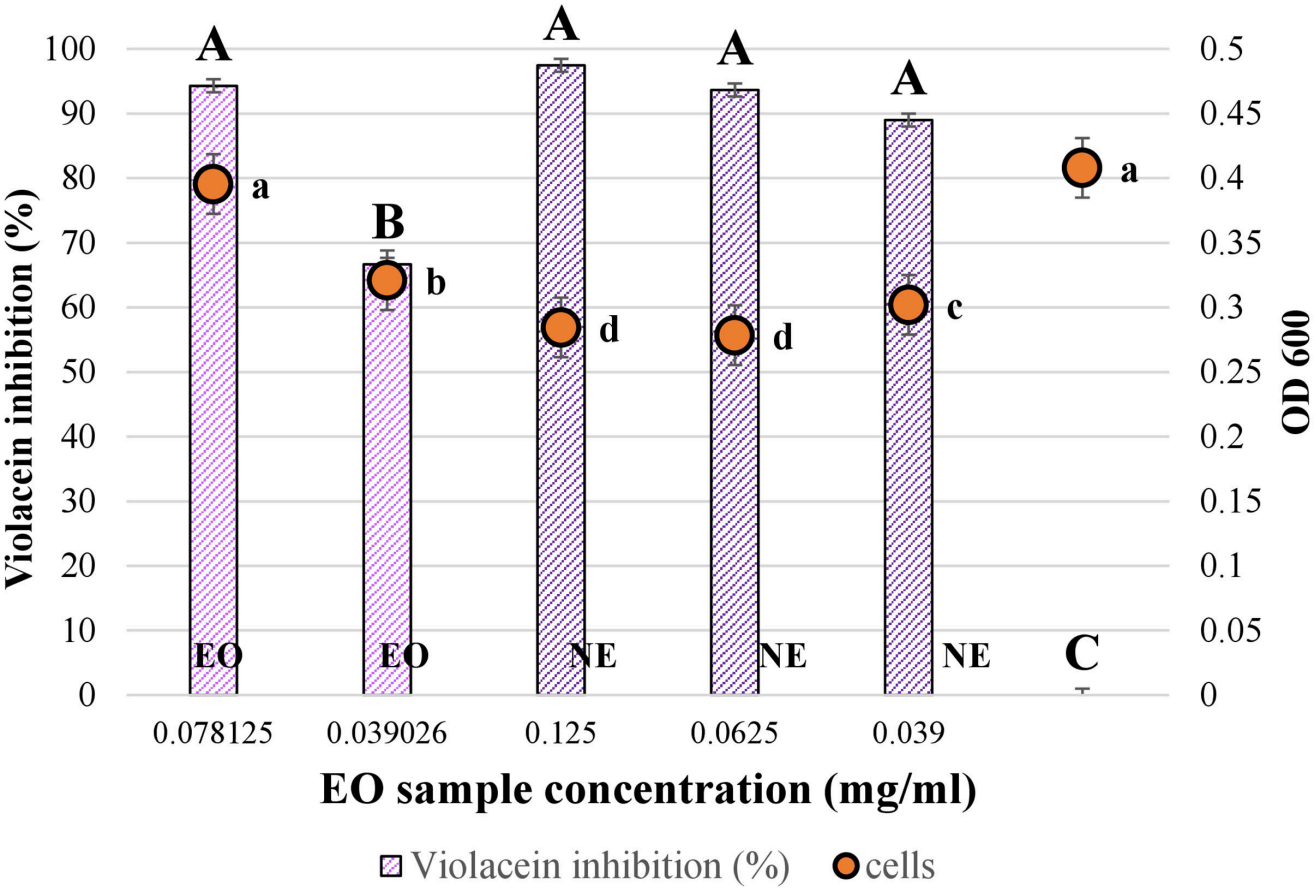
Quorum sensing inhibition assay of oregano essential oil (EO) and oregano nanoemulsion (NE) against *Chromobacterium violaceum*. Percentage of violacein inhibition (%) by at different concentrations and evaluation of microbial viability (OD600) after 36-h incubation in the presence of the OE and NE. Bars and points labeled with different letters indicate significant differences (*p* < 0.05).

In a different study, where cumin and fennel oil emulsions were tested as QS inhibitors by disc diffusion method, 50 μl exhibited anti-QS activity through violacein inhibition around the discz. Those emulsions showed the immediate zone of clearance, causing bactericidal effect followed by the opaque, halo zone of clearance, which indicated the inhibition of violacein production (65). Comparable results were observed in this research, where a slight decrease in the cell viability was observed when the NE was applied as a QS inhibitor (*p* < 0.05). However, a concentration as low as 0.039 mg/ml reduced cell growth but inhibited nearly 90% cell communication (Figure 4). These data suggested that AHL synthesis was probably altered and violacein inhibited by the presence of EONE3 in a dose-dependent manner. Contrary, the cell viability of *C. violaceum* showed no significant difference among control and cultures treated with carvacrol (*P* ≥ 0.05). Carvacrol inhibited the production of violacein product of QS, indicating its interference with QS systems (63). The QS inhibition with original systems like this NE can be an innovative, fresh technique to control food spoilage and to reduce the number of antimicrobials in food.

## Conclusion

Physically stable oil-in-water NEs can be produced using OEO and MCT at different MCT/OEO concentrations. As the OEO concentration increases, the NE droplet size and PDI decreases. EONE3 (160 mg/G NE) is the most stable NE, which shows the smallest droplet size and PDI value after storage. The viscosity of the NE increases, as the concentration of OEO increases, leading to the shear-thinning behavior of the NEs. This effect can be attributed to the presence of OEO terpenes that may solubilize in the palisade layer and change the curvature of the micellar surface, leading to the formation of a WLM structure. Increasing the OEO concentration induces a predominantly solid-like viscoelastic behavior. For EONE3, a weak gel structure can be prepared. EONE presented higher antimicrobial activity than pure EO. Furthermore, a reduction in the intensity of violet pigment produced by *C. violaceum* and no effect on cell growth at concentrations lower than 0.125 mg EO/ml was produced, suggesting that QS in this gram-negative model might be inhibited.

This study provides information about the stability and viscosity and helps to understand the viscoelastic behavior of a NE when the EO concentration varies. These OEO NEs can be used as a novel food preservation technique, reducing cell-to-cell communication (QS) of gram-negative bacteria to lessen food deterioration.

## Data Availability Statement

The raw data supporting the conclusions of this article will be made available by the authors, without undue reservation.

## Author Contributions

CA designed the experiments, did the experimental part, data analyzing, and manuscript writing. PQ did data analyzing and manuscript writing of the rheological behavior study section. AA-G taught and collaborated with CA in the antimicrobial activity and quorum sensing inhibition experiments. QH advised and taught CA during the interneship where experiments where carried out. NG supported the study and revised and corrected the manuscript. All authors contributed to the article and approved the submitted version.

## Conflict of Interest

The authors declare that the research was conducted in the absence of any commercial or financial relationships that could be construed as a potential conflict of interest.
